# Genome-Wide Identification, Classification and Expression Analysis of the *HSP* Gene Superfamily in Tea Plant (*Camellia sinensis*)

**DOI:** 10.3390/ijms19092633

**Published:** 2018-09-05

**Authors:** Jiangfei Chen, Tong Gao, Siqing Wan, Yongheng Zhang, Jiankun Yang, Youben Yu, Weidong Wang

**Affiliations:** College of Horticulture, Northwest A&F University, Yangling 712100, China; jfchen@nwafu.edu.cn (J.C.); gaotong@nwafu.edu.cn (T.G.); wansiqing@nwafu.edu.cn (S.W.); zhangyongheng@nwafu.edu.cn (Y.Z.); jiankunyang@nwafu.edu.cn (J.Y.); chyyjs@nwsuaf.edu.cn (Y.Y.)

**Keywords:** tea plant, HSP, heat stress, drought stress, expression profile

## Abstract

Heat shock proteins (HSPs) function as molecular chaperones. These proteins are encoded by a multigene family whose members play crucial roles in plant growth, development and stress response. However, little is known about the *HSP* gene superfamily in tea plant. In this study, a total of 47 *CsHSP* genes were identified, including 7 *CsHSP90*, 18 *CsHSP70*, and 22 *CssHSP* genes. Phylogenetic and composition analyses showed that CsHSP proteins in the same subfamily have similar gene structures and conserved motifs, but significant differences exist in the different subfamilies. In addition, expression analysis revealed that almost all *CsHSP* genes were specifically expressed in one or more tissues, and significantly induced under heat and drought stress, implying that *CsHSP* genes play important roles in tea plant growth, development, and response to heat and drought stress. Furthermore, a potential interaction network dominated by CsHSPs, including HSP70/HSP90 organizing protein (HOP) and heat shock transcription factor (HSF), is closely related to the abovementioned processes. These results increase our understanding of *CsHSP* genes and their roles in tea plant, and thus, this study could contribute to the cloning and functional analysis of *CsHSP* genes and their encoded proteins in the future.

## 1. Introduction

Plants in the growth stage often have difficulty avoiding threats from various environmental stresses, such as drought, cold, heat, salinity, and heavy metal stress [1,2]. Generally, plants are unable to change their location to escape the abovementioned stresses, but have instead developed various defense mechanisms, including activating the synthesis of heat shock proteins (HSPs), to resist them [3,4]. HSPs are ubiquitous proteins found in plant cells, which were originally detected in plant response to heat stress but are now known to be induced by various biotic and abiotic stresses [5]. HSPs, as molecular chaperones involved in the cellular processes of protein folding and re-folding, assembly, translocation, and degradation, help to maintain the stability of proteins and membranes under stress conditions [6,7,8]. Plant HSPs are divided into five conserved families according to their molecular weights, namely, the HSP100, HSP90, HSP70, HSP60, and small HSP (sHSP) families [9,10]. Among them, the members of the HSP90, HSP70, and sHSP families are the most abundant, and the structures and biological functions of these proteins have received increasing attention [10,11]. Previous studies have confirmed a very high degree of conservation in the structures of HSPs in these three families. HSP90 consists of an N-terminal ATP-binding domain, a C-terminal dimerization domain, and a middle domain that contains an amphipathic loop [12], and HSP70 also contains three distinct domains: an N-terminal ATPase domain, a substrate binding domain, and a highly variable C-terminal domain, and is considered to be the most highly conserved HSP [13]. Notably, HSP110 proteins, a class of macromolecular HSPs, are also included in the HSP70 family, due to their high sequence and structural homology to Hsp70 proteins [14]. In comparison, sHSPs share only a conserved carboxyl-terminal domain of approximately 90 amino acid residues called the a-crystallin domain (ACD), which is characterized by a compact β-strand structure and contains two conserved regions (CRs): CRI with β2, β3, β4, and β5; and CRII with β7, β8, and β9, and a β6 loop [15].

At present, the functional mechanisms of HSP90, HSP70, and sHSP in plant stress response have been widely reported. For instance, the expression of Arabidopsis *HSP90* genes is strongly induced under heat, cold, salinity, and heavy metal stress [16,17]; the overexpression of alfalfa *MsHSP70* and horsegram *MuHSP70* improved the tolerance of transgenic plants to multiple stresses, such as heat, cold, drought, salinity, and oxidative stress [18,19]. In addition, existing studies have confirmed that HSP70 and HSP90 proteins can interact with each other, depending on co-chaperone molecules, such as HOP (HSP70/HSP90 organizing protein), ROF (FK506-binding protein), and HIP (HSP70 interacting protein), to form a complex to jointly respond to external stress [20,21,22]. For example, AtHOP3 interacted with HSP90 and HSP70 to regulate the response of Arabidopsis to different biotic and abiotic stresses [23]. Similarly, the roles of sHSP in plant response to stress have been generally confirmed; for example, rice OsHSP26 could significantly enhance the tolerance of tall fescue to oxidative and heat stresses by protecting photosystem II (PSII) to maintain photosynthesis [24], and a substantial and durable increase in thermotolerance was achieved through sHSP overexpression in hybrid poplar [25]. Furthermore, HSP activity is widely believed to be controlled and regulated by specific types of transcription factors called heat shock transcription factors (HSFs), which not only regulate the expression of *HSP* genes, but also participate in the protein interactions among HSPs [26,27]. To date, the *HSP* gene families have been investigated in several plant species, such as Arabidopsis [17], rice [28], soybean [29], populus [30], and grapes [31], and the functional mechanisms of HSPs in plant stress response have gradually become a hot topic of study.

Tea plant (*Camellia sinensis* (L.) O. Kuntze) is one of the most important economic crops in the world, its young leaves are processed to prepare a popular non-alcoholic beverage known as “tea”, which has positive effects such as delaying ageing and preventing cancer and cardiovascular diseases [32,33]. Tea plant is often affected by harsh environments, and heat and drought stress are important factors that significantly constrain the yield and quality of tea products [34]. Therefore, clarifying the mechanisms of tea plant response to heat and drought stress and identifying resistance genes are of great significance. Recently, four *sHSP* genes in tea plant were reported to play important roles in the process of tea plant response to heat stress [35,36]. However, other studies of HSPs in tea plant are extremely rare. The genome of tea plant has been sequenced and published, rendering the identification and characterization of the *CsHSP* gene superfamily easy and reliable [37]. In this study, 47 *CsHSP* genes were identified, and a comprehensive analysis was performed, including sequence characteristics, phylogenetic relationships, gene structures, conserved motifs, and functional interaction network analysis. In addition, the expression patterns of the *CsHSP* genes were detected in different tissues of tea plants and under heat and drought stress. The results of this study reveal the molecular characteristics of the *CsHSP* gene superfamily and provide a theoretical basis for future studies of the biological functions of CsHSPs under abiotic stresses.

## 2. Results

### 2.1. Identification and Characterization of the CsHSP Gene Superfamily in Tea Plant

A total of 47 *CsHSP* genes were isolated from the tea plant genome and transcriptome database, and their corresponding amino acid sequences were confirmed using the NCBI BLAST program (Version 2.4.0) (Table S1). Among them, 7 (14.89%), 18 (38.29%) and 22 (46.82%) members were determined to belong to the HSP90, HSP70, and sHSP families, respectively. The results of physiological and biochemical properties analysis showed that the lengths of the CsHSP90 proteins ranged from 643 (CsHSP90-7) to 823 (CsHSP90-2) amino acids, the molecular weights were between 73.68 kDa (CsHSP90-7) and 94.32 kDa (CsHSP90-2), and the isoelectric point (pI) values ranged from 4.84 (CsHSP90-2) to 5.20 (CsHSP90-1); the lengths of the CsHSP70 proteins ranged from 407 (CsHSP70-17) to 892 (CsHSP70-3) amino acids, the molecular weights were between 45.45 kDa (CsHSP70-17) and 98.88 kDa (CsHSP70-3), and the pI values ranged from 5.03 (CsHSP70-7) to 9.04 (CsHSP70-17); the lengths of the CssHSPs ranged from 136 (CssHSP-1) to 255 (CssHSP-5) amino acids, the molecular weights were between 15.45 kDa (CssHSP-1) and 28.07 kDa (CssHSP-5), and the pI values ranged from 4.83 (CssHSP-15) to 9.81 (CssHSP-11). More detailed information, including the grand average of hydropathicity (GRAVY), instability index, aliphatic index and subcellular localization, is listed in Table 1.

**Figure 1:**
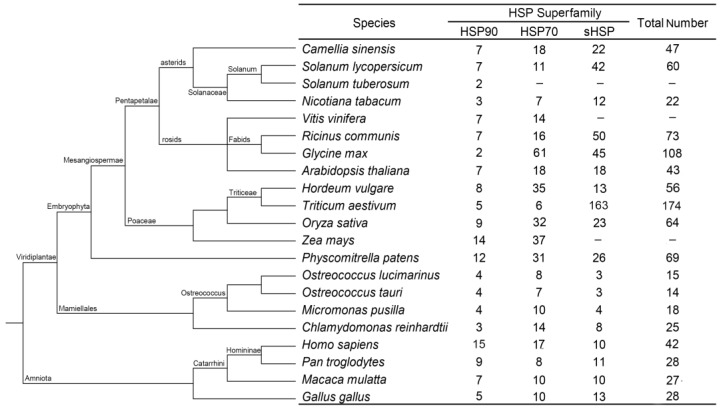
Summary of the *HSP* (heat shock protein) gene superfamily among 21 species.

Twenty other species in which *HSP* superfamily genes have been identified were comparatively analyzed to further evaluate the family composition of the *CsHSPs*, and the results showed that the distribution of the *CsHSP* members maintained a similar proportion to those of other higher plants, namely, the HSP90 family had significantly fewer members than the HSP70 and sHSP families did. However, the above trend is not evident in lower plants, which have very few *sHSP* members. In addition, the total number of *HSPs* in lower plants is significantly less than that in higher plants. In animals, almost equal numbers of genes were found in the three HSP families, showing a distribution clearly different from those of higher plants (Figure 1).

### 2.2. Phylogenetic Analysis of CsHSPs

**Figure 2:**
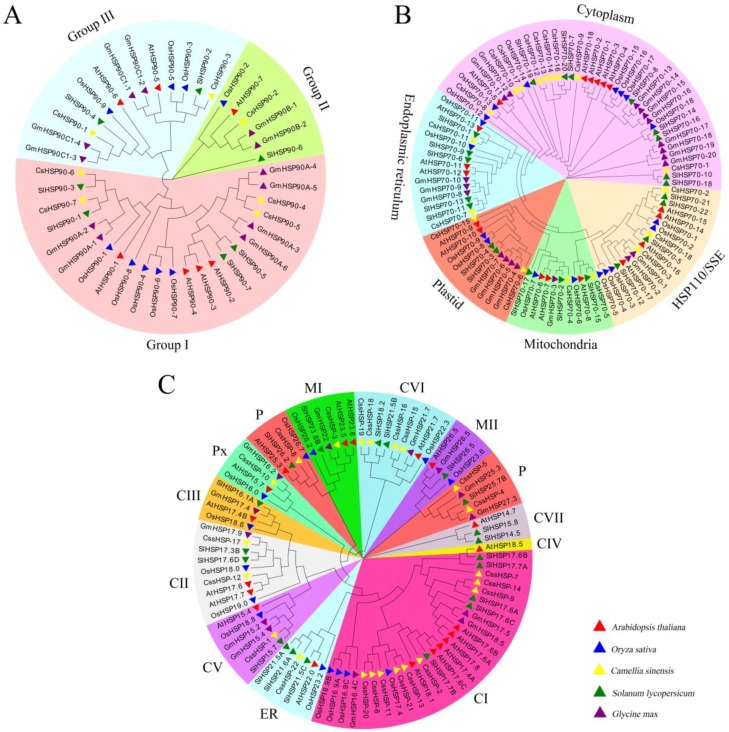
Phylogenetic tree of CsHSP90 (**A**), CsHSP70 (**B**), and CssHSP (**C**). Phylogenetic tree of HSP proteins in tea plant and other plant species were generated by MEGA 7 using neighbor-joining. Cs: *Camellia sinensis*, At: *Arabidopsis thaliana*, Os: *Oryza sativa*, Gm: *Glycine max*, and Sl: *Solanum lycopersicum*; CI–CVII: cytoplasm I–VII, ER: endoplasmic reticulum, MI–MII: mitochondria I–II, P: plastid, and Px: peroxide. The protein ID of all species is listed in Table S2.

By clustering the HSP90 proteins of Arabidopsis, rice, tomato, soybean, and tea plant, we found that the CsHSP90 proteins were divided into 3 groups. Group I included 4 members (CsHSP90-4, -5, -6, -7), while group II and group III contained 1 member (CsHSP90-2) and 2 members (CsHSP90-1 and CsHSP90-3), respectively (Figure 2A). Similarly, the CsHSP70 proteins were classified into 5 subfamilies, including 9 in the cytoplasm (CsHSP70-1, -8, -9, -10, -12, -13, -14, -16, and -17), 2 in the ER (CsHSP70-7 and CsHSP70-11), 2 in the mitochondria (CsHSP70-6 and CsHSP70-15), 2 in the plastid (CsHSP70-4 and CsHSP70-5), and 3 in the HSP110/SSE (CsHSP70-2, -3 and-18) subfamily (Figure 2B). According to the classification of sHSPs in Arabidopsis [38], the 22 CssHSPs were classified into only 9 protein subfamilies, including CI (9), CII (2), CV (1), CVI (4), CVII (1), ER (1), MI (1), P (3), and Px (1), while no CssHSPs existed in the CIII, CIV, and MII subfamilies (Figure 2C). Interestingly, the P subfamily clustered into two branches, and was closely related to the MI and MII subfamilies, suggesting that they have similar origins and evolutionary relationships.

### 2.3. Gene Structures of the CsHSP Gene Superfamily 

**Figure 3:**
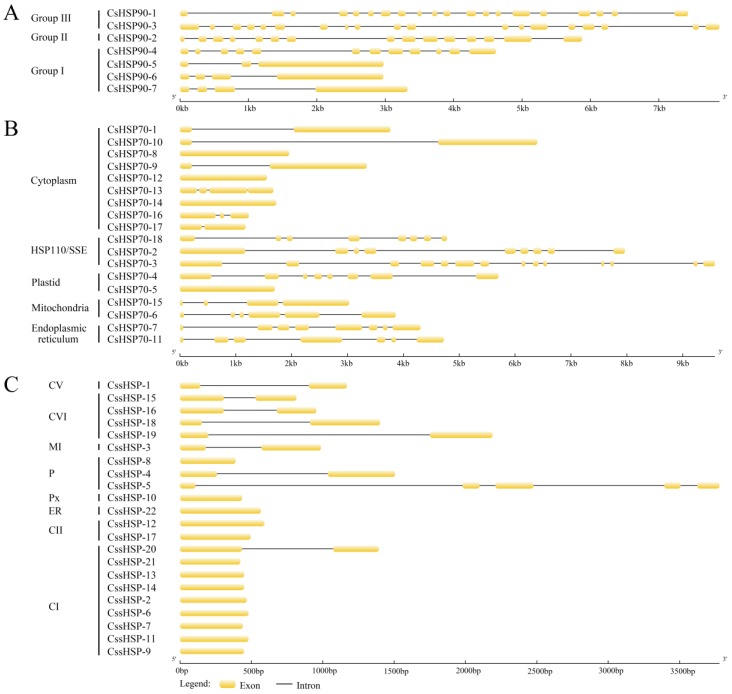
Exon–intron structure analyses of *CsHSP90* (**A**), *CsHSP70* (**B**), and *CssHSP* (**C**) genes. The yellow sections represent exons, and the grey parts indicate introns. CI, CII, CV, and CVI: cytoplasm I, II, V and VI; ER: endoplasmic reticulum, MI: mitochondria I; P: plastid; and Px: peroxide.

To further analyze the evolutionary relationships of the *CsHSP* gene superfamily, exon–intron diagrams of the *CsHSP90*, *CsHSP70*, and *CssHSP* genes were generated according to their genome and coding sequences. Group II and III *CsHSP90* genes are characterized by a large number of introns (14–19), while group I *CsHSP90* genes contain only a few introns (2–3), except for *CsHSP90-4* that has 11 introns (Figure 3A). In the *CsHSP70* genes, there are 0–3 introns in the cytoplasm subfamily genes, 3–5 introns in the mitochondria subfamily genes, and at least 6 introns in the ER, P, and HSP110/SSE subfamily genes (Figure 3B). In comparison, most *CssHSP* genes have no introns or one intron; only *CssHSP-5*, in the P subfamily, contains 4 introns (Figure 3C).

### 2.4. Conserved Protein Motif Analysis of CsHSPs

**Figure 4:**
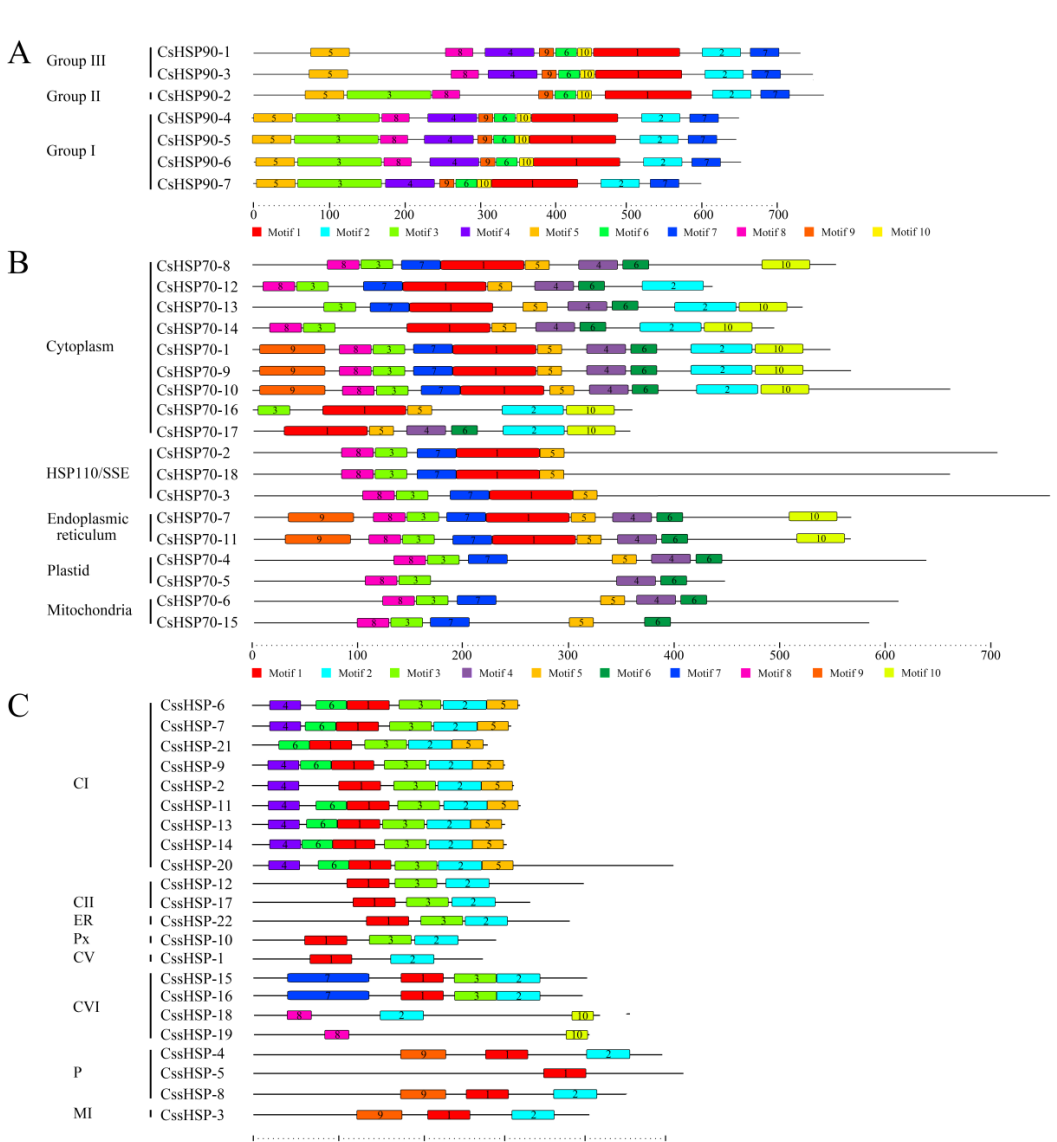
Distribution of conserved motifs in CsHSP90 (**A**), CsHSP70 (**B**), and CssHSP (**C**). Putative motifs are represented by a number in a colored box. CI, CII, CV, and CVI: cytoplasm I, II, V and VI; ER: endoplasmic reticulum, MI: mitochondria I; P: plastid, and Px: peroxide.

To investigate the protein sequence features of the CsHSPs, 10 motifs in the CsHSP90, CsHSP70, and CssHSP families were individually predicted by the MEME tool, and the regular expression levels of the conserved motifs are listed in Table S3. In the CsHSP90 family, all members have a similar motif composition, although small differences between members in different group are also present (Figure 4A). In the CsHSP70 and CssHSP families, members belonging to the same subfamily contain similar motifs, while the type and number of motifs in the different subfamilies of CsHSP70s and CssHSPs differ significantly (Figure 4B,C). In addition, protein motifs 1, 2, and 3 in the CssHSPs are highly conserved and distributed across almost all members; together, they form the ACD protein domain of the sHSP (Figure S1), which is the structural basis for the biological function of sHSPs.

### 2.5. Protein Interaction Network of CsHSPs

**Figure 5:**
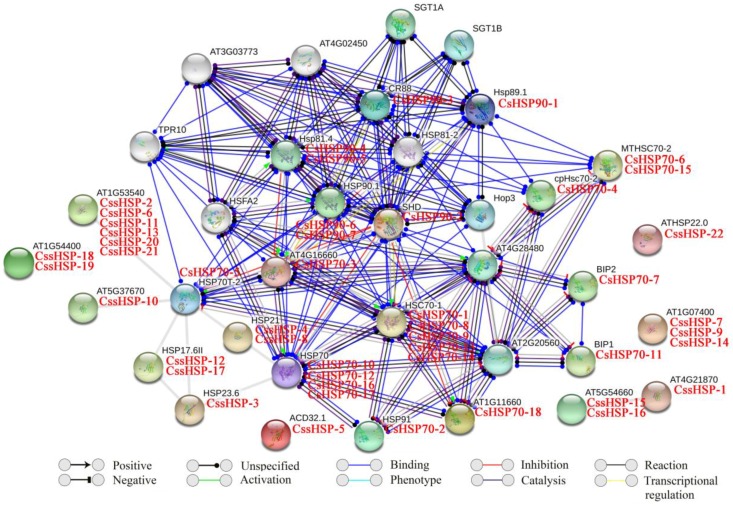
Putative interaction network of CsHSPs in tea plant. Homologous proteins in tea plant and Arabidopsis are shown in red and black, respectively.

In this study, the functional and physical protein interactions of the CsHSPs were validated by constructing an Arabidopsis association model using STRING software (version 10.5, https://string-db.org/). The results showed that 47 CsHSPs associated with 26 known Arabidopsis HSPs participated in the interaction network (Table S4 and Figure 5). As expected, the network showed general and complex interactions between HSP70 and HSP90 family proteins; for example, HSP70 (corresponding to CsHSP70-10, -12, -16, and -17), HSP70T-2 (corresponding to CsHSP70-5), HSC70-1 (corresponding to CsHSP70-1, -8, -9, -13, and -14) and MTHSC70-2 (corresponding to CsHSP70-6 and -15) closely interact with HSP81.4 (corresponding to CsHSP90-4 and -5), SHD (corresponding to CsHSP90-2), and HSP90.1 (corresponding to CsHSP90-6 and -7). Simultaneously, HOP3, an HSP70/HSP90 organizing protein, was closely connected to many HSP70 and HSP90 family proteins, implying that it plays important roles in their functions. In addition, 3 HSP110/SSE subfamily proteins (corresponding to CsHSP70-2, -3, and -18) showed very positive interactions with other HSP70 family proteins, especially the members of the cytoplasm subfamily. Furthermore, an extensive interaction was observed between the transcription factor HSFA2 and the HSP70 and HSP90 proteins. By contrast, some sHSPs (corresponding to CssHSP-2, -3, -6, 10, -11, -12, -13, -17, -20, and -21) were associated with only HSP70T-2 proteins, and others were independent.

### 2.6. Expression Patterns of CsHSP Genes in Different Tissues of Tea Plant

**Figure 6:**
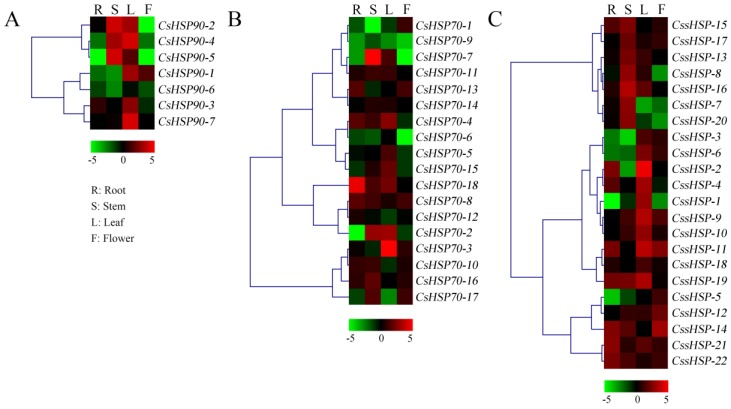
Expression patterns of *CsHSP90* (**A**), *CsHSP70* (**B**), and *CssHSP* (**C**) genes in different tissues of tea plant. R indicates root; S, stem; L, leaf; F, flower. The heatmap was generated by MeV software (TIGR, Rockville, MD, USA) using the *CsHSP* genes’ expression data, and normalized log_2_ transformed values were used with hierarchical clustering. The red and green colors indicate higher or lower transcript abundances than those of the relevant control, respectively.

To elucidate the tissue-specific expression patterns of *CsHSP* genes, qRT-PCR was used to determine the expression levels of 47 *CsHSP* genes in four tissues, including the root, stem, leaf, and flower of tea plant (Figure 6 and Table S5). All *CsHSP90* genes were highly expressed in the leaf, and *CsHSP90-2*, *-4*, and *-5* were also highly expressed in the stem (Figure 6A). Most *CsHSP70* genes are differentially expressed in different tissues, although they have no obvious regularity (Figure 6B). In addition, we found that 7 *CssHSP* genes had higher expression levels in the stem, 10 *CssHSP* genes had higher expression levels in the leaf, and 5 *CssHSP* genes had higher expression levels in the flower, although some of these genes are also significantly expressed in other tissues (Figure 6C). In general, almost all *CsHSP* genes were specifically expressed in one or more tissues, implying that these genes play different roles in the growth and development of tea plant.

### 2.7. Expression Profiles of CsHSP Genes in Response to Heat and Drought Stress

**Figure 7:**
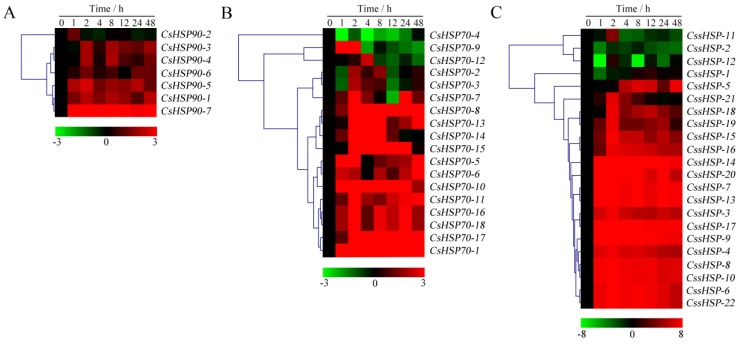
Expression profiles of *CsHSP90* (**A**), *CsHSP70* (**B**), and *CssHSP* (**C**) genes in tea plant under heat stress (40 °C). The heatmap was generated by MeV software using the *CsHSP* genes expression data, and normalized log_2_ transformed values were used with hierarchical clustering. The red and green colors indicate higher or lower transcript abundances than those of the relevant control, respectively.

**Figure 8:**
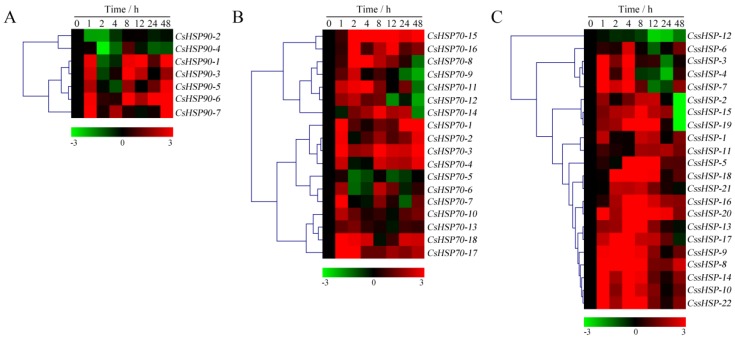
Expression profiles of *CsHSP90* (**A**), *CsHSP70* (**B**), and *CssHSP* (**C**) genes in tea plant under drought stress (20% polyethylene glycol (PEG) 6000). The heatmap was generated by MeV software using the *CsHSP* genes expression data, and normalized log_2_ transformed values were used with hierarchical clustering. The red and green colors indicate higher or lower transcript abundances than those of the relevant control, respectively.

As shown in Figure 7 and Table S6, the expression of all *CsHSP90* genes was significantly upregulated under heat stress, although the expression levels of certain genes briefly and slightly decreased at individual time points (Figure 7A). Among the *CsHSP70* genes, 12 members showed highly upregulated expression throughout the heat treatment process, 5 members were upregulated in the early stage and downregulated in the late stage, and the expression of the *CsHSP70-4* gene was significantly suppressed (Figure 7B). Similarly, the expression of 18 *CssHSP* genes was significantly upregulated and maintained at a very high level, while the remaining 4 *CssHSP* genes, including *CssHSP-1*, *-2*, *-11*, and *-12*, were significantly downregulated, although the expression levels of certain genes briefly increased at 2 h or 24 h (Figure 7C). By contrast, the expression trends of the *CsHSP90, CsHSP70*, and *CssHSP* genes under drought stress were complex and diverse, although most of them also showed upregulated expression (Figure 8 and Table S7). Specifically, most *CsHSP90* genes were downregulated at 2 and 4 h, and significantly upregulated at other time points (Figure 8A). Among the *CsHSP70* genes, the expression levels of 5 members showed a trend of increasing first and then decreasing, and other members were upregulated overall, although the change trends of certain genes were weak at 2 to 24 h (Figure 8B). All *CssHSP* genes, except *CssHSP-12*, were upregulated under drought stress, and the expression of most of them also showed a trend of increasing first and then decreasing (Figure 8C). In addition, the expression trends of most *CsHSP* genes were identical under both drought and heat stress, but the opposite was observed in a few genes, including *CsHSP90-2*, *CsHSP90-4*, *CsHSP70-4*, *CssHSP-2*, and *CssHSP-11* (Figure 7 and Figure 8). These results reveal that almost all *CsHSP* genes are involved in the responses of tea plant to heat and drought stress, and the response mechanisms of different *CsHSP* genes to different stresses are complex and diverse.

## 3. Discussion

HSPs are ubiquitous molecular chaperones that play vital roles during plant growth and development, and protect the plant cellular machinery under stress conditions [5,6,10]. The biological function of HSPs has been widely studied in many plants, such as Arabidopsis [14], rice [28], and wheat [39]. However, only a few efforts have been made to elucidate HSPs in tea plant. In this study, a comprehensive genome-wide analysis of the *CsHSP* gene superfamily in tea plant was carried out, and the results will provide a powerful theoretical foundation for future functional studies.

In the current study, 47 *CsHSP* genes were identified in tea plant, including 7 *CsHSP90*, 18 *CsHSP70*, and 22 *sHSP* genes, demonstrating a similar proportion to the distribution of *HSP* members in other higher plants. Notably, the number of *HSP* genes is much greater in higher plants than in lower plants, which may be because higher plants generally require more genes for biological processes [40]. However, *HSP* gene numbers do not correspond with evolutionary level in higher plants, implying diversity in plant *HSP* gene evolution [41]. It has been reported that the phylogenetic relationships of HSPs are closely correlated with their subcellular localizations, and subfamilies are usually named for their protein localization in this group [9,15]. Our phylogenetic analysis of the CsHSP90, CsHSP70, and CssHSP families showed results highly consistent with the predictions of protein localization, which further confirms the above conclusions. Notably, three CsHSP70 proteins (CsHSP70-2, -3, and -18) were clustered into the HSP110/SSE subfamily, which generally act as nucleotide exchange factors for cytoplasmic HSP70 proteins, and participate in HSP70-mediated protein folding [42], suggesting that CsHSP70-2, -3, and -18 may have similar biological functions. This hypothesis has been confirmed to some extent by the results of the protein interaction network prediction, which showed positive interactions of CsHSP70-2, -3, and -18 with cytoplasmic CsHSP70 proteins. In addition, the 22 CssHSPs were grouped into only 9 subfamilies, fewer than the 12 subfamilies in Arabidopsis and similar to the sHSP classifications in pepper [43], tomato [44], and rice [26]. Intriguingly, the CssHSPs in the P and M (MI and MII) subfamilies were closely related to each other, which was consistent with the sHSPs of the M subfamily having evolved later from the P subfamily [39,43,45].

Previous studies have shown that gene organization plays an important role in the evolution of multiple gene families and in response to stress conditions [46]. In our investigation, we found that the most closely related *CsHSP* genes in the same subfamily share similar gene structures, indicating that gene structures shed light on phylogenetic relationships. In addition, cytoplasmic *CsHSP* genes generally have few introns, whereas organelle-specific *CsHSPs* have more introns, similar to the *HSP* genes in other plants [16,31,43], suggesting that intron pattern is closely related to gene function. Additionally, there are indications that genes that must be rapidly activated in response to stress tend to evolve a decreased intron density [43]. Our results showed that most *CssHSP* genes have no introns or just one intron, which contributes to their transcriptional regulation under stress conditions; this feature provides a possible explanation for how almost all of these *CssHSP* genes are rapidly and strongly induced under heat and drought stress [46]. Furthermore, our conserved motif analysis showed that CsHSPs in the same subfamily contain similar protein motifs but differ significantly among the different subfamilies, especially the members of the CsHSP70 and CssHSP families; this result supports our abovementioned results from the phylogenetic analysis of CsHSPs. Notably, 3 highly conserved protein motifs were detected in the majority of CssHSPs; these motifs form the ACD domain that is the structural basis for the biological function of sHSPs, which is consistent with earlier findings in pepper and switchgrass [43,45]. 

Numerous studies have shown significant histological specificity in the transcription of plant *HSP* genes [13,47]. For example, 11 tomato *sHSP* genes were shown to have tissue- and development-specific expression in the leaf, root, and hypocotyl [44]; the transcription of *AtHSP90-1* could be detected only in roots; and several *AtHSP90* genes are highly expressed in the root, but have very low or no expression in other organs [48]. Similarly, most *PtHSP90* genes in populus are mainly expressed in the stem [12], while the *OsHSP90* genes of rice are specifically expressed in different tissues [49]. In the present study, we also found that almost all *CsHSP* genes were specifically expressed in one or more tissues, especially the leaf and stem of tea plant, similar to the results from recent reports in rice [50], tomato [44], and switchgrass [49], suggesting that the *CsHSP* genes are extensively involved in the growth and development of tea plant, and may play different roles in different tissues. The name of the HSP family is well known to be derived from their rapid and efficient expression under heat stress, which has been widely confirmed in various plant species [9,11,13]. As expected, all *CsHSP90* genes and most *CsHSP70* and *CssHSP* genes were significantly induced under heat stress, and the expression levels of the *CssHSP* genes were maintained at an extremely high level, similar to the results of earlier investigations in other plants [44,51,52], suggesting that CsHSPs are critical in tea plant response to heat stress. On the other hand, more recent studies have indicated that HSPs also play important roles in plant responses to other biotic and abiotic stresses in addition to heat stress, especially drought stress [4,30]. For example, five switchgrass *sHSP* genes were significantly upregulated under severe drought stress [45], and some *HSP70* and *HSP90* genes are also induced by drought stress in rice and populus [30,49,51]. Our results showed that most genes in the *CsHSP90*, *CsHSP70*, and *CssHSP* families also showed upregulated expression under drought stress, although their expression trends were complex and diverse, implying that they actively participated in the drought stress response of tea plant. In addition, we found differences in the response mechanisms of different *CsHSP* genes to different stresses, implying a diversity of CsHSP functions in the stress response mechanisms of tea plant [45].

Previous investigations have confirmed that HSPs interact with each other to regulate plant growth, development, and stress response [13,53]. HSP90 and HSP70 interact, depending on the action of their co-chaperone molecules, to form a complex to jointly respond to external stress [20,21,22]. In the present study, we also found that the interaction between CsHSP90 and CsHSP70 family proteins is ubiquitous, and may play important roles in tea plant responses to heat and drought stress. Simultaneously, we speculated that HOPs participate in the above process as the co-chaperones connecting the CsHSP90 and CsHSP70 proteins, similar to the recent reports that HOP interacting with HSP90 and HSP70 regulated stress responses in Arabidopsis and orchardgrass [54,55]. In addition, our data confirm that HSP110/SSE subfamily proteins positively interact with cytoplasm subfamily HSP70 proteins, as previously demonstrated in both plants and yeast [56,57]. In addition, some CssHSPs were also found to interact with a few CsHSP70s, similar to previous reports in pea and tobacco [58,59]; thus, they may cooperate to act in the stress response of tea plant. Furthermore, numerous studies have confirmed that the expression of *HSP* genes is regulated by HSF transcription factors, such as Arabidopsis HSPA2, and populus HSFs transcriptionally regulate the expression of different *HSP* genes participating in plant development and response to abiotic stresses [60,61]. HSFs have also been confirmed to be involved in the protein interactions of HSPs in plants, which are vital in plant response to multiple stresses [60,61]. Our results showed that most CsHSP90 and CsHSP70 members were closely related to HSFA2, implying that CsHSPs are also regulated by HSFs; this regulation may include the transcript and protein levels. Overall, these results suggest that the interaction network dominated by CsHSP, including HOP and HSF, is ubiquitous and plays important roles in tea plant growth, development and stress response. 

In conclusion, this study provides the first comprehensive and systematic analysis of the HSP gene superfamily in tea plant. In total, 47 *CsHSP* genes from the *HSP90*, *HSP70*, and *sHSP* families were identified, and bioinformatics and expression profile analyses of these genes were performed to determine their potential functions in the growth, development, and stress responses of tea plant. The results indicate that *CsHSP* genes are actively involved in regulating tea plant growth, development and responses to heat and drought stress, and these processes are closely related to the interaction network involving CsHSP, HOP, and HSF. These results provide an important foundation for further functional studies investigating the CsHSPs and contribute to illuminating the mechanisms of stress tolerance in tea plant.

## 4. Materials and Methods

### 4.1. Plant Materials, Growth Conditions, and Stress Treatments

Cutting seedlings of 2-year-old tea plant (*C. sinensis* cv. “*Longjingchangye*”) were grown in a chamber at Northwest A&F University (Yangling, China) under a 12 h light (300 μmol·m^−2^·s^−1^)/12 h dark cycle at 23 ± 2 °C ambient temperature and 70 ± 5% relative humidity. Then, the tea plants were exposed to heat and drought stress, respectively. For heat treatment, the tea plants grown in normal conditions were transferred to an artificial climate chamber maintained at 40 °C for 48 h. For drought treatment, the root of tea plants together with the medium was immersed completely in the solution containing 20% (*w*/*v*) polyethylene glycol (PEG) 6000 for 48 h. All stress treatments were completed under other consistent growth conditions. The first or second tender leaves of the treated tea plants were respectively collected at 0, 1, 2, 4, 8, 12, 24, and 48 h, immediately frozen in liquid nitrogen, and then stored at −80 °C for further analysis.

### 4.2. Identification of the CsHSP Gene Superfamily in Tea Plant

The amino acid sequences of the HSPs of *Arabidopsis thaliana* and other plants were downloaded from the TAIR (http://www.arabidopsis.org/) and NCBI databases; the detailed information is shown in Table S2. All the candidate *CsHSP* genes of tea plant were retrieved from the tea plant genome [37,62] and transcriptome (NCBI SRA: SRP128078) database. The amino acid sequences of the AtHSPs were selected as query sequences to identify the CsHSPs. The online program SMART (http://smart.embl-heidelberg.de/) and the NCBI Conserved Domain Database (http://www.ncbi.nlm.nih.gov/Structure/cdd/wrpsb.cgi) were used to survey the conserved domains of the candidate proteins.

### 4.3. Sequence Analysis and Phylogenetic Tree Construction 

The exon–intron structures of the *CsHSP* genes were displayed through Gene Structure Display Server 2.0 (http://gsds.cbi.pku.edu.cn). The sequences of the CsHSPs were analyzed with ExPASy ProtParam (http://www.expasy.org/tools/protparam.html) to obtain the number of amino acids, molecular weight, theoretical isoelectric point (pI), and instability index. The WoLF PSORT program (https://wolfpsort.hgc.jp/) was used to predict the subcellular localization of CsHSPs. Multiple sequence alignment of CsHSPs with other plants’ HSP proteins (Table S2) were performed using DNAMAN 6.0. Unrooted neighbor-joining phylogenetic trees were constructed using MEGA 7.0 and EvolView (http://www.evolgenius.info/evolview).

### 4.4. Conserved Motif Analysis and Protein Interaction Network Prediction of CsHSPs 

The MEME program (version 4.10.0, http://meme-suite.org/) was used to identify the conserved protein motifs of all CsHSPs, with the following parameters: number of repetitions any, maximum number of motifs 10, and optimum motif widths from 6 to 200 amino acid residues. The functional interacting network models of CsHSPs were integrated in STRING (https://string-db.org/) with the confidence parameter set at a 0.40 threshold.

### 4.5. RNA Isolation and Expression Analysis of CsHSP Genes

Total RNA from tea plant leaves and other tissues was extracted using the Plant RNA Kit (Omega, Norcross, GA, USA), and the concentration and integrity of the RNA samples were checked by a NanoDrop ND 1000 spectrophotometer (Thermo Fisher Scientific, Waltham, MA, USA) and 1.2% agarose gel electrophoresis. Subsequently, 1 μg of total RNA was reverse transcribed to first-strand cDNA by the 5× All-In-One RT MasterMix Kit (ABM, Richmond, BC, Canada) according to the manufacturer’s protocol, and the cDNA was diluted 10-fold for standby. Quantitative real-time PCR (qRT-PCR) was performed using SYBR^®^ Premix Ex Taq™ II (TaKaRa, Dalian, China) on an iQ5 real-time PCR platform (Bio-Rad, Hercules, CA, USA). Briefly, each reaction was performed in a total volume of 20 µL containing 10 µL SYBR^®^ Premix Ex Taq II, 1 μL diluted cDNA template, 0.8 μL each primer, and 7.4 μL ddH_2_O using the following PCR program: 95 °C for 3 min, followed by 40 cycles of 95 °C for 30 s and 60 °C for 1 min 30 s. Melting curves were obtained to verify the amplification specificity through a stepwise heating of the amplicon from 60 to 95 °C. Primer pairs (Table S8) were designed by OligoAnalyzer 3.1 (https://sg.idtdna.com/calc/analyzer) and tested by NCBI Primer BLAST. The *CsPTB* gene was used as an internal control gene [63]. Three independent biological replicates were performed, and the relative expression levels of the *CsHSP* genes were calculated with the 2^−∆∆*C*t^ method [64]. The heatmap was created using MultiExperiment Viewer (MeV). 

## Figures and Tables

**Table 1:** Summary information of physiological and biochemical properties of the CsHSP proteins.

Name	Unigene ID	Animo Acids	MW (kDa)	pI	GRAVY	Instability Index	Aliphatic Index	Subcellular Localization
*CsHSP90-1*	TEA014732.1	790	89.90	5.20	−0.58	34.37	77.62	mito: 7, chlo: 4, nucl: 2, cysk: 1
*CsHSP90-2*	TEA027510.1	823	94.33	4.84	−0.75	37.01	79.62	E.R.: 14
*CsHSP90-3*	TEA017408.1	810	92.31	5.01	−0.60	43.77	80.26	chlo: 11.5, chlo_mito: 7.5, mito: 2.5
*CsHSP90-4*	TEA027510.1	701	80.44	5.11	−0.60	35.08	84.95	cyto: 7, nucl: 2, plas: 2, mito: 1, vacu: 1, golg: 1
*CsHSP90-5*	TEA008112.1	699	79.99	4.99	−0.56	36.68	84.23	cyto: 8, plas: 2, E.R.: 2, mito: 1, golg: 1
*CsHSP90-6*	TEA007238.1	707	81.14	5.04	−0.59	42.75	82.26	nucl: 6, cyto: 5, chlo: 2, cysk: 1
*CsHSP90-7*	TEA027790.1	643	73.68	5.11	−0.50	39.33	85.99	cyto: 9, nucl: 3, plas: 1, golg: 1
*CsHSP70-1*	TEA005970.1	648	71.03	5.16	−0.42	34.56	81.87	cyto: 7, cysk: 6, chlo: 1
*CsHSP70-2*	TEA029156.1	846	93.33	5.21	−0.42	44.20	80.18	cyto: 8, chlo: 2, nucl: 2, cysk: 2
*CsHSP70-3*	TEA023352.1	892	98.89	5.22	−0.46	41.33	83.55	E.R.: 14
*CsHSP70-4*	TEA003665.1	709	75.51	5.38	−0.29	33.70	85.51	chlo: 14
*CsHSP70-5*	TEA016684.1	569	61.81	5.54	0.09	45.27	100.39	cyto: 11, chlo: 2, nucl: 1
*CsHSP70-6*	TEA022542.1	679	73.09	6.02	−0.33	41.82	86.07	mito: 12, chlo: 2
*CsHSP70-7*	TEA023884.1	669	73.73	5.03	−0.45	31.40	86.43	E.R.: 14
*CsHSP70-8*	CL5657.Contig2_All▲	651	71.19	5.20	−0.45	34.66	80.06	cyto: 12, chlo: 1, nucl: 1
*CsHSP70-9*	TEA025820.1	677	75.12	5.54	−0.27	34.56	87.70	plas: 11, nucl: 1, vacu: 1, E.R.: 1
*CsHSP70-10*	TEA024518.1	744	82.42	5.79	−0.43	32.96	82.34	cyto: 8, cysk: 5, chlo: 1
*CsHSP70-11*	TEA023111.1	669	74.02	5.14	−0.42	31.86	87.43	plas: 11.5, golg_plas: 7, golg: 1.5, E.R.: 1
*CsHSP70-12*	CSA029398.1◆	519	57.32	6.15	−0.31	39.76	88.86	cysk: 11, chlo: 2, cyto: 1
*CsHSP70-13*	TEA014652.1	614	67.30	5.43	−0.37	31.36	85.94	chlo: 9, nucl: 1, cyto: 1, extr: 1, vacu: 1, pero: 1
*CsHSP70-14*	TEA024522.1	574	63.62	7.99	−0.43	37.81	85.45	cyto: 11, chlo: 2, nucl: 1
*CsHSP70-15*	TEA029776.1	627	69.61	5.07	0.01	36.23	106.67	cyto: 9, chlo: 2, nucl: 2, cysk: 1
*CsHSP70-16*	TEA029256.1	449	49.87	5.32	−0.52	40.99	79.73	extr: 6, cyto: 3, chlo: 2, vacu: 1, E.R.: 1, mito_plas: 1
*CsHSP70-17*	TEA024511.1	407	45.46	9.04	−0.38	32.41	87.00	chlo: 11, mito: 1, extr: 1, E.R.: 1
*CsHSP70-18*	TEA017380.1	767	85.85	5.60	−0.41	43.64	83.42	nucl: 6, chlo: 3, cyto: 2, cysk: 2, vacu: 1
*CssHSP-1*	TEA023567.1	136	15.46	5.60	−0.18	58.91	88.16	cyto: 10, chlo: 1, nucl: 1, extr: 1, golg: 1
*CssHSP-2*	TEA025531.1	155	17.42	5.13	−0.57	52.60	80.52	cyto: 13, chlo: 1
*CssHSP-3*	TEA030322.1	199	22.45	5.99	−0.56	52.92	88.14	mito: 11, chlo: 3
*CssHSP-4*	TEA028966.1	243	27.40	8.29	−0.71	43.78	62.55	chlo: 13, nucl: 1
*CssHSP-5*	TEA031630.1	255	28.08	8.76	−0.43	60.82	69.25	chlo: 10, nucl: 2, extr: 2
*CssHSP-6*	TEA004366.1	159	18.25	5.68	−0.70	62.21	71.70	cyto: 14
*CssHSP-7*	TEA031692.1	153	17.47	5.53	−0.61	54.38	73.20	cyto: 14
*CssHSP-8*	TEA033542.1	223	25.19	5.83	−0.53	46.89	81.26	chlo: 10, mito: 2, vacu: 2
*CssHSP-9*	TEA015431.1	150	17.25	5.56	−0.71	44.10	68.20	cyto: 13, extr: 1
*CssHSP-10*	CL9235.Contig2_All▲	144	16.08	6.76	−0.49	41.09	83.12	pero: 8, nucl: 2, cyto: 2, chlo: 1, golg: 1
*CssHSP-11*	CSA020588.1◆	159	18.34	9.81	−0.56	45.22	83.96	cyto: 6, mito: 5, nucl: 3
*CssHSP-12*	CSA025700.1◆	196	21.66	5.30	−0.71	52.67	68.67	cyto: 8, nucl: 6
*CssHSP-13*	TEA032865.1	149	17.05	8.83	−0.72	39.98	69.93	cyto: 14
*CssHSP-14*	TEA015431.1	149	17.11	5.38	−0.65	44.27	68.66	cyto: 7, nucl: 3, extr: 2, chlo: 1, plas: 1
*CssHSP-15*	TEA010603.1	198	22.77	4.83	−0.58	46.14	69.34	chlo: 5, nucl: 5, cyto: 3, plas: 1
*CssHSP-16*	TEA021291.1	195	22.15	5.29	−0.59	43.43	70.36	nucl: 5, cyto: 5, chlo: 3, plas: 1
*CssHSP-17*	TEA033850.1	164	18.08	5.78	−0.37	39.30	83.84	cyto: 8, nucl: 5, chlo: 1
*CssHSP-18*	TEA005909.1	205	22.91	9.52	−0.41	32.13	84.73	cyto: 3, golg: 3, plas: 2.5, E.R.plas: 2.5, mito: 2, E.R.: 1.5, chlo: 1, vacu: 1
*CssHSP-19*	TEA005935.1	199	20.95	8.93	−0.28	47.34	76.58	E.R.: 3.5, golg: 3, cyto: 2.5, E.R.plas: 2.5, mito: 2, cyto_pero: 2, chlo: 1, vacu:1
*CssHSP-20*	TEA004364.1	249	27.72	9.49	−0.43	48.28	74.90	mito: 9, nucl: 3, chlo: 2
*CssHSP-21*	TEA028346.1	140	16.25	6.77	−0.88	42.55	74.43	cyto: 5, extr: 4, nucl: 2, golg: 2, plas: 1
*CssHSP-22*	TEA017741.1	188	21.78	6.47	−0.49	49.13	92.82	chlo: 7, mito: 3, cyto: 2, extr: 1, E.R.: 1

Note: Sequenced IDs marked with triangle are from transcriptome, with diamond are from *C. sinensis* var. *assamica* (CSA) genome, others are from *C. sinensis* var. *sinensis* (CSS) genome. chlo: chloroplast, cyto: cytoplasm, ER: endoplasmic reticulum, extr: extracellular, golg: golgi apparatus, mito: mitochondria, nucl: nucleus, pero: peroxide, plas: plasma membrane, vacu: vacuole.

## Supplementary Materials

Supplementary materials can be found at http://www.mdpi.com/1422-0067/19/9/2633/s1.
