# Supplementary material for: Genome-Wide Identification, Classification and Expression Analysis of the HSP Gene Superfamily in Tea Plant (Camellia sinensis)
Source: Int J Mol Sci. 2018 Sep 5;19(9):2633. doi: 10.3390/ijms19092633 (PMC6164807; doi:10.3390/ijms19092633)
Supplement: Supplementary file 1 [file ijms-19-02633-s001.zip › ijms-347277-supplementary-final/Table S3.docx]

**Table S3.** Regular expression of conserved motifs of CsHSP proteins.

| **Protein** | **Motif** | ***E* value** | **Width** | **Site** | **Best Possible Match** |
| --- | --- | --- | --- | --- | --- |
| CsHSP90 | 1 | 1.40E-293 | 125 | 7 | ENKEDY[NK]KF[YW]E[AN]F[SG]KNLKLG[IC][HI]EDS[TQ]N[RHK][AKT][KR]LA[DEP]LLR[YF][HF]S[TS]KSGDE[ML][TI]SL[DK]DYV[TE][RN]MKEGQ[KS]DIYYI[TA]G[ED]SKK[AS][VA]ENSPFLE[KR]L[KL][KE]K[GD][IY]EVL[YF][ML][VI]D[AP]IDE[YV]A[VI][GQ][QN]LKE[YF][EDK][GE]KK[LF]V[SD][AI][TS]KE[GD]LKL[DG][DE] |
|  | 2 | 5.90E-185 | 57 | 7 | [VQ]VS[DN]R[LIV][VS]DSPC[CV][LV]V[TS]G[EK][YF]GW[TS]ANMER[IL]MKAQ[AT]L[RG]D[ST]S[MS][ALS][EGS][YF]M[SR][SG][KR][KR][TV][ML]EINP[DE][NH][PG]I[MI] |
|  | 3 | 2.60E-229 | 123 | 5 | [QN][PA][EK]L[FD]I[QRH]I[KVI][PL][DN]K[ETV][NK][KN][TI][LI][ST]I[IR]D[SR]GIGMTK[AE]DL[VI][NK]NLGTIA[RK]SGT[KS][EA]F[MV]E[AK][LM][QAL][AT][GS][AGS]D[VL][SN][ML]IGQFGVGFYS[AV]YLVA[DE][KRY]V[IEV]V[TVI][ST]KHN[DE]D[EK][QR]Y[VI]WES[QK]A[GD]G[SA]F[TA][VI][TRS][RE]D[TV][TDNW][GN]E[PSQ]LGRGT[KE][IMV][TR]L[YFH]L[KR][ED][DE][QA] |
|  | 4 | 4.60E-167 | 73 | 6 | KKK[IV]KE[VK][SY][HW][ED]W[ES]L[IV]N[KE][QT]KPIW[LM]R[KN]P[EK]E[IV]TKEEY[ANS][AE]FYK[SK][LT][TF]N[DE]W[EL][ED][HP]LAVKHFS[VT]EG[QE]LEF[KR][AS]IL[FY]VPK[RM]APF |
|  | 5 | 1.10E-152 | 57 | 7 | M[AST][EN][AT]E[KT][FY][EA][FY]QAE[VI][SN][RQ]L[LM][DS][LI]I[IV]N[ST][LF]YSNK[ED][IV]FLREL[IV]SNASDALDK[IL]RF[LE]S[LV]TD[KP][SE][KIL]L |
|  | 6 | 1.30E-89 | 31 | 7 | EL[IF]P[ER]YL[SG]FVKG[VI]VDS[DEN]DLPLN[IV]SRE[MI]LQ[QE][NS] |
|  | 7 | 5.00E-74 | 41 | 7 | S[VA]K[DR][LA]V[DLM]LL[YF][ED]TAL[LI][TS]SGF[ST][LP]DDP[NS]T[FL][GA][NA][RK]I[HY][RE]M[LM]KL[GA]LS[IG] |
|  | 8 | 3.80E-55 | 40 | 6 | E[YF]LE[EP]RR[LI]K[DE]LVK[KN][HY]S[EQ]F[IV]S[FY]PI[YS][LT]W[IQ]EKTT[ET][KV]E[IV][SE]DDED |
|  | 9 | 8.50E-53 | 21 | 7 | [TV][RN]KK[MPT][NK]NI[KR]LYV[RK]RVFI[MS]D[ND][CF] |
|  | 10 | 5.40E-30 | 21 | 7 | I[LV][KR][VI][IM]RK[NR]LV[KR]K[CA][FIV][ED][ML][FI][FN]EI[AS] |
| CsHSP70 | 1 | 1.9e-747 | 113 | 13 | V[QA][EQ]FKRK[HY]KKDISGNPR[AS]LRRLRTACE[RK]AKR[TI]LS[SA]T[AT]Q[TA]TIEI[DE]SL[YF][DE]G[IV]DFY[TS][TP]ITRARFEE[LM]NMDLFRK[CT]MEPVEK[CA]LRDA[KG][ML]DK[SN][SN][VI]H[DE][VI]VLVGGSTRIPKVQQLLQDFF |
|  | 2 | 1.80E-247 | 57 | 8 | KYKSEDEEHKKKVEAKNALENYAYNMRNT[IV]KDEKI[SG][AT]KL[PS][PF]ADKKKIEDAIDQAI[HQ]W |
|  | 3 | 3.10E-241 | 33 | 17 | ATKDAGVIAGLNV[ML]R[IL]INEPTAAA[IL]AYGLDKK[AG] |
|  | 4 | 6.60E-277 | 41 | 13 | GGVMT[VK]LIPRNTTIPTKK[ES]QVF[ST]T[YA]SDNQ[PT]GVLIQVYEGER |
|  | 5 | 9.90E-208 | 29 | 17 | GKE[LP]CKS[IV]NPDEAVAYGAA[VL]Q[AG][AG]ILS[GD][ED]G |
|  | 6 | 1.10E-202 | 29 | 14 | NNLLGKFEL[TS]GIPPAPRGVPQI[EN]V[CT]FD[IV]D |
|  | 7 | 2.10E-252 | 41 | 15 | GGGTFDVS[ILV]L[TE][IF][EDS][EN]G[IV]F[EK]V[KL][AS]T[AS]GDTHLGGEDFDN[RV][ML]V[NE][HY]F |
|  | 8 | 2.00E-222 | 41 | 15 | F[AST][PA]E[EQ][IV][SL][AS]M[VI]L[IT]K[ML]KE[IT]AE[AKS][YF]LG[KT][TKP][VI]K[NDK]AV[IV]T[VI]PAYFND[SA]Q |
|  | 9 | 1.90E-201 | 98 | 5 | [GK][EL]G[PT][AV]IGIDLGTTYSCVGV[WY][QKR][HN][DG][HR]VEIIANDQGNR[TI]TPS[YW]V[AG]FTD[TS]ERLIG[DE]AAKNQ[VA]A[MLV]NP[ETI][NR]T[VI]FD[AVT]KRLIGR[RK][FY][SDET]D[AKP][SE]V[QV][SKR]D[MIT][KE]L[WAL]P[FY]K[VI][IV][ANP][GK][PDE]G |
|  | 10 | 1.10E-165 | 41 | 10 | D[ADS]NQLAE[AK][DE]EFE[DEY][KN][MV]KELE[SAC][IV]CNPII[AT]K[MV]YQG[AS]GG[GA]D[MG][GA][GP]A |
| CssHSP | 1 | 3.20E-203 | 26 | 20 | DWKETP[EN][AS][HY][VI][FL]KAD[LM]PG[LV]KKE[ED][VI]KVE |
|  | 2 | 5.00E-188 | 26 | 20 | [FY][LM]RRF[RE]LPEN[AV][KD][VM][DE][KE][IV]KAS[MV][EK][ND]GVLT |
|  | 3 | 9.70E-118 | 26 | 15 | LQISGER[KS][KRV]EKEE[KE][NT]D[KT]WHR[VI]ERSSG |
|  | 4 | 1.30E-51 | 19 | 8 | [FI][FG][GN][GRS][RQ][GR]S[NS][IV]FDPFSLD[IP]W[DR] |
|  | 5 | 7.30E-43 | 19 | 9 | VT[VI]PK[EV][EK][VE]KKPE[VI]K[SA]I[DE]IS |
|  | 6 | 1.10E-27 | 19 | 8 | [LN][FA][NP][FI][SP][EI][SF][AS][RQ]E[TN]S[AQ][FI]AN[AT]R[IV] |
|  | 7 | 2.20E-14 | 50 | 2 | KWCVPMTE[DG]VF[EG]TL[IM]S[KT][GS]SP[MT][AQ]HTVFGDGSLFSPLLFGKFFDPSDAFPLW |
|  | 8 | 4.70E-02 | 15 | 2 | [MN]PPYE[DE]FEPLC[GN]W[QR][HR] |
|  | 9 | 5.70E-01 | 28 | 3 | [IP][GD][LV][LW]D[PR][FL][SP][PT][AMT]R[AST][LMV][QRS]Q[ML][LM][DEN][LRT]M[DE][RQ][FLM][MF]E[DN][PS][FV] |
|  | 10 | 7.20E-01 | 14 | 2 | [AC]LGAY[AI][MT]Y[KS][CF]RSL[DY] |
