# Supplementary figures and images for: Genome-Wide Identification, Classification and Expression Analysis of the HSP Gene Superfamily in Tea Plant (Camellia sinensis)
Source: Int J Mol Sci. 2018 Sep 5;19(9):2633. doi: 10.3390/ijms19092633 (PMC6164807; doi:10.3390/ijms19092633)

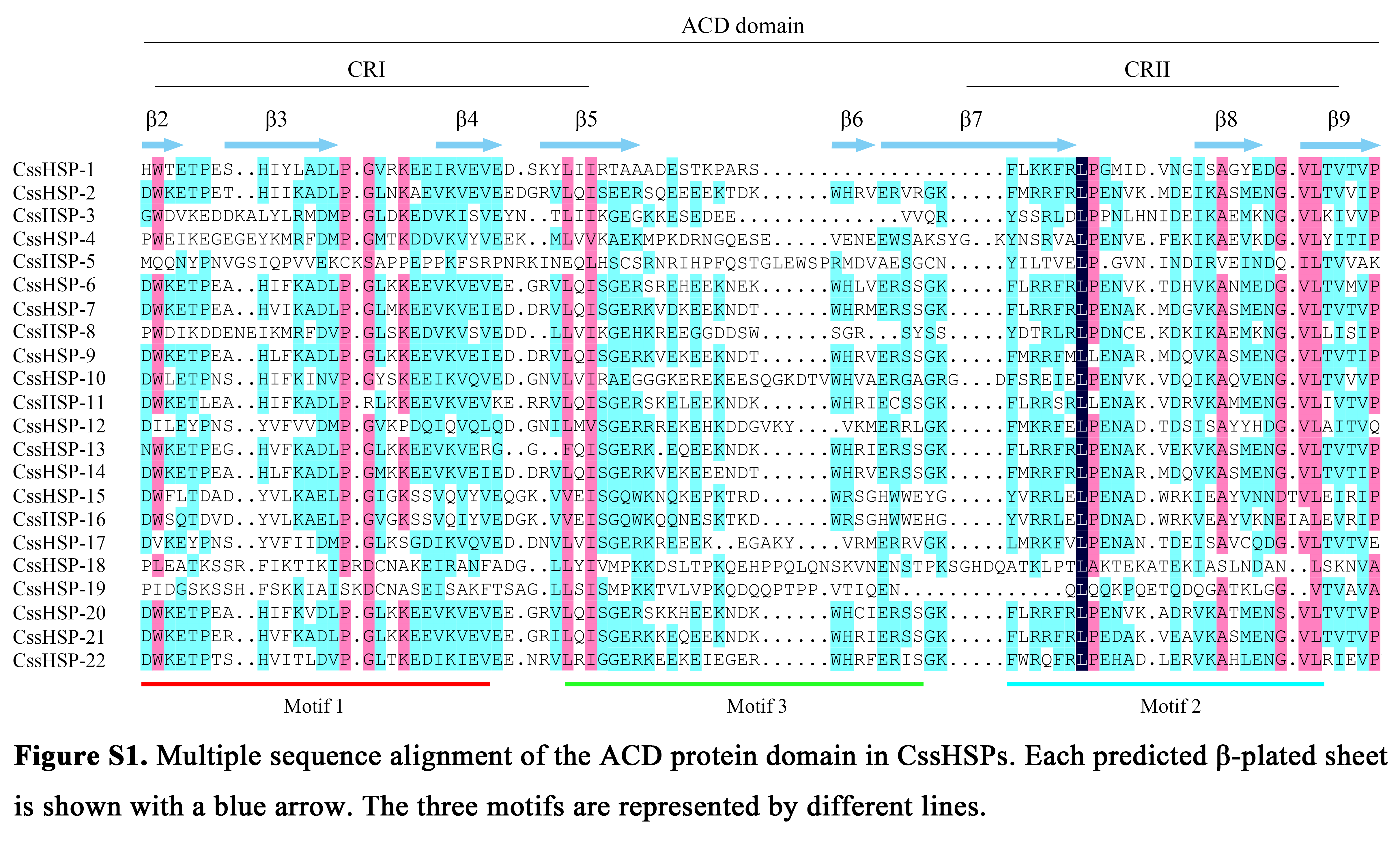

Supplement: Supplementary file 1 [file ijms-19-02633-s001.zip › ijms-347277-supplementary-final/Figure S1.tif]
